# Personalized Surgical Strategies in Breast Cancer: Preliminary Evaluation of the Alexis^®^ Retractor for Reducing Postoperative Complications and Improving Operative Efficiency

**DOI:** 10.3390/jcm14217688

**Published:** 2025-10-29

**Authors:** Lorenzo Scardina, Enrico Di Guglielmo, Cristina Accetta, Beatrice Carnassale, Sabatino D’Archi, Alba Di Leone, Annasilvia Di Pumpo, Flavia De Lauretis, Antonio Franco, Federica Gagliardi, Stefano Magno, Francesca Moschella, Maria Natale, Eleonora Petrazzuolo, Chiara Rianna, Alejandro Martin Sanchez, Marta Silenzi, Gianluca Franceschini

**Affiliations:** Breast Unit, Department of Women, Children and Public Health Sciences, Fondazione Policlinico Universitario Agostino Gemelli IRCCS, 00168 Rome, Italy; lorenzo.scardina@policlinicogemelli.it (L.S.);

**Keywords:** Alexis^®^ retractor, axillary surgery, breast cancer, postoperative complications, breast surgery

## Abstract

**Background**: Personalized medicine in breast cancer surgery aims to tailor therapeutic strategies not only to tumor biology but also to patient-specific risk factors and surgical outcomes. The Alexis^®^ retractor, originally designed for abdominal and pelvic surgery, may represent an innovative tool to optimize axillary surgical procedures in selected patients. Its atraumatic design and protective sheath provide enhanced visibility, minimized tissue trauma, and a potentially lower risk of postoperative complications, thus contributing to individualized surgical care. **Methods**: We conducted a retrospective, single-center study at Fondazione Policlinico Universitario Agostino Gemelli IRCCS between January 2024 and April 2025. Patients undergoing breast-conserving surgery or mastectomy with axillary access were included. The Alexis^®^ retractor was used for axillary tissue retraction in procedures such as sentinel lymph node biopsy and axillary dissection. Outcomes were assessed at 7, 14, and 30 days postoperatively, with particular focus on complication rates and surgical efficiency. **Results**: Thirty-seven patients (38 procedures) were analyzed. Seromas occurred in four patients (10.8%) and were managed with ultrasound-guided aspiration. Wound dehiscence occurred in two patients (5.4%) and was treated with advanced dressings. No infections, hemorrhages, or flap necrosis were observed. No systemic complications occurred. **Conclusions**: The preliminary results suggest that the Alexis^®^ retractor may support a more personalized approach to axillary surgery in breast cancer, by reducing early postoperative complications and improving surgical ergonomics. Its atraumatic design and protective sheath may help tailor surgical management to individual patient risk profiles, minimizing tissue damage and infection risk while enhancing intraoperative visibility and efficiency. Further prospective, controlled studies with larger cohorts are needed to confirm its role in precision breast surgery and to define which patient subgroups may benefit the most.

## 1. Introduction

Minimizing tissue trauma and reducing postoperative complications are essential priorities in modern breast surgery, especially for procedures involving axillary access, such as sentinel lymph node biopsy (SLNB) and axillary lymph node dissection (ALND). In the age of personalized medicine, surgical approaches are increasingly customized not only to tumor biology and systemic treatments but also to individual patient factors, comorbidities, and risk profiles. Improving surgical tools to lower morbidity and enhance recovery is thus a vital part of precision breast cancer care.

Traditional rigid metal retractors, while effective in maintaining exposure, have been associated with several drawbacks, including mechanical tissue stress, nerve or vascular injury, and impaired wound healing, which can compromise both oncologic outcomes and patient recovery [1,2]. Such complications are particularly relevant in patients with a higher individual risk of surgical morbidity (e.g., obesity, diabetes, smoking, or previous irradiation), where minimizing tissue trauma is essential to deliver safer, more individualized treatment.

The Alexis^®^ wound retractor has emerged as an innovative surgical device offering an atraumatic alternative to conventional retractors. Composed of a soft, flexible polymer sheath anchored by internal and external rings, the Alexis^®^ provides circumferential and self-retaining retraction that conforms to patient anatomy, ensuring consistent exposure while minimizing localized pressure and mechanical trauma [3,4]. This design has demonstrated benefits across various surgical disciplines, including colorectal, gynecologic, and abdominal procedures, where its use has been linked to reduced surgical site infections, improved visualization, and enhanced procedural efficiency [5,6,7].

In the context of breast cancer surgery, particularly in axillary approaches, the need for precise dissection within confined anatomical spaces underscores the value of optimized surgical access. The Alexis^®^ retractor enhances intraoperative visibility by providing optimal exposure of critical structures while maintaining stable wound edges. Unlike metal retractors, which may cause minor tissue trauma and ischemia, the Alexis^®^ reduces the risk of complications such as seroma, hematoma, wound dehiscence, and flap necrosis [8,9]. Furthermore, by preserving microcirculation and reducing compressive forces, the device may also mitigate postoperative pain and support faster recovery [10].

An additional clinical advantage is the potential for reduced infection risk. The retractor’s smooth, non-porous polymer surface minimizes bacterial adherence and protects wound margins, thereby maintaining a sterile field and reducing contamination—an especially relevant concern in oncologic procedures [11,12,13].

Despite its increasing use in general surgery, limited data are currently available on the role of the Alexis^®^ retractor in breast oncology. In particular, few studies have evaluated its impact on surgical outcomes in axillary procedures for breast cancer.

The aim of this study is to evaluate the effectiveness and safety of the Alexis^®^ retractor during axillary surgery in breast cancer patients, with a specific focus on its potential role in personalized surgical care. We share our single-center experience, highlighting short-term postoperative complications, surgical visibility, and intraoperative efficiency and emphasizing its possible function in customizing surgical management to each patient’s profile.

## 2. Materials and Methods

### 2.1. Study Design and Setting

This retrospective, single-center study was conducted at the Fondazione Policlinico Universitario Agostino Gemelli IRCCS, Rome, Italy, over a 16-month period from January 2024 to April 2025. The study was approved by the local institutional ethics committee and all patients provided informed consent for surgical procedures and data use. All consecutive patients undergoing oncologic breast surgery with axillary incision, either breast-conserving surgery (BCS) or mastectomy, were screened for eligibility. Inclusion criteria comprised female patients aged ≥18 years requiring axillary lymph node evaluation by either SLNB or ALND. Exclusion criteria included previous axillary surgery, active infection, or an allergy to device materials. In all enrolled cases, the Alexis^®^ wound retractor (Applied Medical, Rancho Santa Margarita, CA, USA), size XXS, was used during the axillary procedure. The device was employed to provide continuous, circumferential, and atraumatic retraction of the axillary wound, replacing traditional metal retractors.

### 2.2. Device Placement Technique

**Figure 1:**
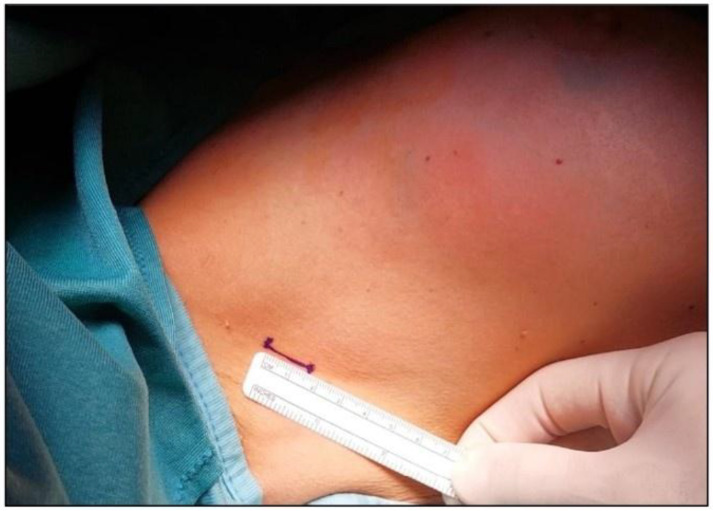
To ensure optimal placement of the Alexis^®^ wound retractor, an incision no larger than 1.5 cm is required.

**Figure 2:**
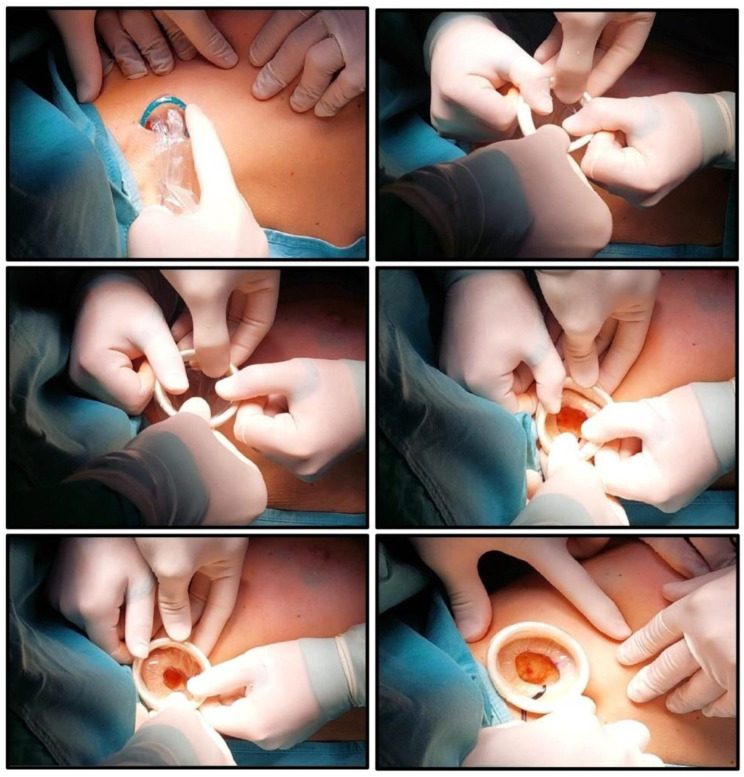
The inner ring is inserted into the surgical wound. The outer ring is unrolled to cover the wound edges. The outer ring is progressively tensioned and folded onto itself until it contacts the skin, ensuring stable retraction.

**Figure 3:**
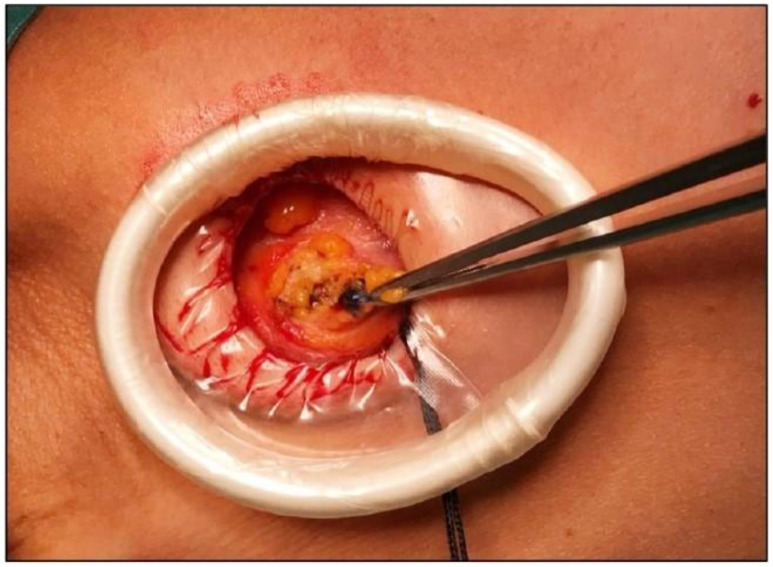
Intraoperative identification of sentinel lymph node.

**Figure 4:**
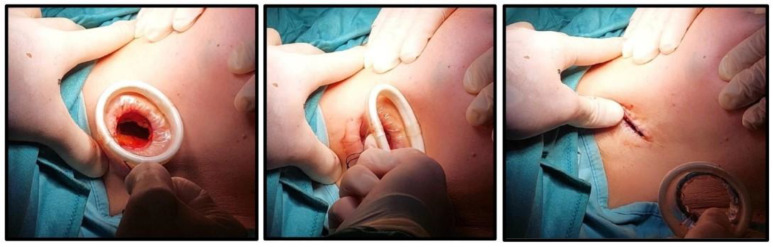
Device removal.

A standard axillary skin incision was made with the length strictly limited to 1.5 cm to ensure optimal stability of the internal rigid ring (Figure 1). After blunt dissection of subcutaneous flaps, the internal ring of the Alexis^®^ was inserted into the prepared pocket, ensuring that the retrieval suture was oriented inferiorly to facilitate later removal. Subsequently, the external soft ring was gradually rolled inward until it lay flush against the skin surface, creating a secure and self-retaining exposure of the axillary cavity (Figure 2 and Figure 3). This setup eliminated the need for additional manual retractors, thereby enhancing surgical visibility and access. At the end of the procedure, the device was removed by applying gentle traction to the retrieval suture (Figure 4), followed by thorough palpation of the wound to confirm complete lymph node excision. At the end of the procedure, a suction drain was placed in the axilla only in the two patients who underwent ALND, in accordance with institutional practice. No drains were used in cases of SLNB.

### 2.3. Surgical Protocol and Follow-Up

All procedures were performed by experienced breast surgeons from the multidisciplinary breast unit, using standardized surgical protocols. Postoperative follow-up visits were scheduled at 7, 14, and 30 days post-surgery. At each time point, patients underwent clinical evaluations for early postoperative complications, including:

- Surgical site infection
- Seroma (clinically evident or confirmed by ultrasound)
- Hematoma
- Wound dehiscence
- Skin flap necrosis

### 2.4. Data Collection and Analysis

Demographic and clinical variables were prospectively recorded including age, body mass index (BMI), smoking status, neoadjuvant treatment and type of breast surgery (Table 1). The primary outcome was the rate of early surgical complications.

## 3. Results

A total of 37 patients undergoing oncologic breast surgery with axillary access were included in the study, resulting in 38 axillary procedures due to one bilateral case. The median patient age was 59 years (range: 37–80), and the mean body mass index (BMI) was 25.09 kg/m^2^. In terms of smoking history, 14 patients (37.8%) were current smokers, 4 (10.8%) were former smokers, and 19 (51.4%) had never smoked (Table 1). None of the patients included in the study had a diagnosis of diabetes mellitus or other major chronic metabolic conditions.

Of the 38 axillary procedures performed, 33 (86.8%) involved SLNB and 5 (13.2%) involved ALND. Seven patients (18.9%) had received neoadjuvant chemotherapy (NACT) prior to surgery.

During the 30-day follow-up period, early surgical complications were observed in 7 procedures (18.4%). Specifically, axillary seroma occurred in four patients (10.8%), including one bilateral case, yielding a total of five seroma events (13.1% of procedures). All seromas were managed conservatively via ultrasound-guided aspiration in an outpatient setting. Additionally, two patients (5.4%) developed wound dehiscence, which was successfully treated with advanced local wound care.

No cases of surgical site infection, hematoma, or skin flap necrosis were recorded. No cases of nerve injury or persistent postoperative numbness were observed in the study population. Furthermore, no systemic complications occurred in any patient during the observation period.

Notably, all complications occurred following SLNB procedures; no complications were observed among patients undergoing ALND. None of the patients who experienced complications had received NACT. Among the six procedures associated with complications, four were performed in active smokers, and two involved patients with a BMI >25 kg/m^2^. These findings may suggest a potential association between smoking status and increased risk of postoperative wound complications. Conversely, neither neoadjuvant treatment nor the extent of axillary surgery (SLNB vs. ALND) appeared to correlate with a higher incidence of complications in this cohort.

Surgeons consistently reported enhanced visualization and stable exposure of the axillary cavity when using the Alexis^®^ retractor. In all cases, the device provided adequate self-retaining retraction without the need for additional manual retractors. This contributed to a simplified operative workflow, minimizing the number of instruments required and facilitating surgical precision.

## 4. Discussion

The findings of this preliminary study indicate that using the Alexis^®^ retractor in axillary breast cancer surgery may reduce early postoperative complications and provide practical benefits in surgical exposure and workflow. No cases of surgical site infection, hematoma, or flap necrosis were reported, and all observed complications, which were limited to seroma formation and wound dehiscence, were minor and managed conservatively in an outpatient setting.

Seroma was the most commonly observed complication, consistent with previous literature that identifies it as the leading postoperative issue following axillary procedures [14]. Interestingly, all complications occurred in patients who underwent SLNB, while none were noted after ALND. Although this may seem surprising, it probably reflects the small sample size rather than an actual inverse relationship between surgical extent and complication risk. The absence of seroma in the ALND group should therefore be viewed with caution, as only five patients had ALND, rendering the study underpowered to determine the true incidence of procedure-specific complications. This limitation highlights the need for larger comparative studies to better evaluate complication rates in this subset.

The absence of complications among patients who received NACT aligns with emerging data suggesting that modern systemic treatments, when combined with careful surgical techniques, do not significantly hinder wound healing [15]. In contrast, most complications were seen in active smokers, consistent with well-established evidence that tobacco use adversely affects tissue oxygenation, immune response, and wound integrity [16]. Although the study was not designed to detect statistical links, this trend highlights the importance of including preoperative smoking cessation counseling as part of a personalized perioperative plan. Elevated BMI was observed in two of the six patients with complications, indicating a possible impact of individual metabolic factors; however, the small subgroup size prevents definitive conclusions [17].

Although variables such as NACT, smoking status, and BMI are known to influence postoperative outcomes, subgroup analysis based on these factors was not feasible in the present study due to the limited sample size. Nevertheless, these observations highlight how patient-specific characteristics can significantly affect surgical risk, emphasizing the importance of customizing surgical approaches within a personalized medicine framework.

Therefore, the absence of complications in the ALND group is almost certainly a reflection of the very small number of patients and should not be interpreted as the device being more protective in more extensive surgeries.

The complication rate observed in our series with the Alexis^®^ retractor appears comparable or better than those reported in the literature with conventional retractors. Notably, the incidence of seroma in our group was lower than the usually reported rates, which range from 15% to 49% after ALND with standard techniques [18]. We documented no cases of surgical site infection or nerve injury, whereas literature reports infection rates of 5–10% and transient or permanent sensory disturbances in up to 30% of cases [19]. These findings suggest that using atraumatic devices could improve surgical safety and support risk-adjusted strategies, especially for patients with a higher baseline risk of complications.

From a technical perspective, the Alexis^®^ retractor appeared to offer excellent visualization and stable, self-retaining exposure of the axillary cavity. These features helped reduce the need for additional manual retractors and enhanced operative efficiency. Similar advantages have been reported in other surgical fields, including colorectal [20,21] and orthopedic surgery [22], where the device has been linked to lower infection rates and better wound management. In breast surgery, the ability to maintain stable exposure while minimizing trauma may lead to more personalized operative strategies, especially for patients where gentle tissue handling is essential (e.g., following neoadjuvant therapy or in high-risk metabolic conditions).

In the current era of precision medicine and personalized surgery, especially in breast oncology, ongoing innovation in surgical techniques and device choices is crucial [23,24]. The Alexis^®^ retractor exemplifies such innovation, providing improvements in surgical exposure, tissue protection, and potentially aesthetic results [8,9]. Beyond its clinical usefulness, the Alexis^®^ also supports a minimally invasive approach: using an ultra-small incision (≤1.5 cm) yielded excellent cosmetic results with almost invisible scarring. Although aesthetic benefit was not a predefined goal of the study, it became a notable observation, particularly in personalized breast cancer care, where patient satisfaction, body image, and quality of life are key outcomes [25].

In line with our focus on personalized surgical strategies, the use of the Alexis^®^ retractor was individually tailored preoperatively, with the anticipated axillary staging (cN0) as the primary driver of selection. Candidates were identified through clinical examination and targeted axillary ultrasound (with sampling when indicated), particularly when a SLNB-only pathway was expected. This approach clarifies how feasibility and safety were embedded in a personalized decision-making process rather than a one-size-fits-all device adoption.

An important observation was the effect of the surgical learning curve. Four of the six complications (66.6%) happened within the first 10 cases, indicating that early technical adaptation may influence outcomes. In the remaining 27 procedures, only two complications (7.4%) occurred. This suggests that, as surgeon experience increases, the Alexis^®^ can be used more effectively and safely. These findings emphasize that device adoption should be tailored to each case, considering both surgeon expertise and patient-specific risk factors.

A known limitation of the Alexis^®^ retractor is the difficulty in repositioning once applied, which may be relevant during ALND, where exposure needs can change. However, with proper incision planning, we found that the device provides sufficient and stable retraction in most cases. We acknowledge that this technique may not be suitable for all patients and may require a learning curve and careful case selection. This further reinforces the concept of personalization, where surgical devices and techniques should be matched to patient anatomy and procedural demands.

A formal cost-effectiveness analysis was not included in this study and falls beyond its original scope. However, the economic implications of routinely adopting the Alexis^®^ retractor remain an important aspect to consider, particularly in relation to potential benefits in operative efficiency, complication reduction, and resource utilization.

Despite its encouraging results, the study is subject to several limitations including its retrospective design, limited sample size, and lack of a control group using conventional retractors. These factors limit the generalizability of the findings and prevent formal conclusions on comparative effectiveness. Prospective, randomized studies are needed to validate the protective and functional benefits of the Alexis^®^ device and to further quantify its impact on long-term outcomes, including operative time, pain control, and quality of life. Importantly, future research should also investigate how patient-specific factors such as BMI, comorbidities, or neoadjuvant treatment can guide the selective use of the Alexis^®^ retractor to maximize benefits within a personalized surgical framework.

## 5. Conclusions

The Alexis^®^ wound retractor seems to be a safe and effective tool in axillary breast cancer surgery. Its atraumatic, self-retaining design offers reliable wound edge protection, improves surgical field visibility, and decreases the need for manual retraction, thus supporting a more efficient and minimally invasive approach.

This study showed that the device is feasible and safe, with a complication rate within the expected range reported in the literature. Additionally, it resulted in smaller incisions and better aesthetic outcomes, supporting modern goals in breast surgical oncology that focus not only on cancer control but also on patient-centered outcomes like recovery, satisfaction, and quality of life.

In personalized medicine, the Alexis^®^ retractor can be a helpful tool for customizing axillary surgery based on each patient’s profile, especially for those with a higher risk of wound complications or where cosmetic outcomes matter most. By reducing tissue damage and enabling risk-based strategies, the device could enhance surgical accuracy and individualization.

While further studies in larger, controlled groups are needed, the Alexis^®^ retractor shows potential as a useful tool to improve surgical quality, efficiency, and cosmetic results and to support the advancement of precision breast surgery.

## Figures and Tables

**Table 1:** Clinical characteristics and complications of 37 breast cancer patients (SLNB: Sentinel Lymph Node Biopsy, ALND: Axillary Lymph Node Dissection, BMI: Body Mass Index, and NACT: Neoadjuvant Chemotherapy).

Characteristics	Number	Procedures with Any Complications	Seroma	Wound Dehiscence
Patients	37	6 (16.2%)	4 (10.8%)	2 (5.4%)
Procedures	38	7 (18.4%)	5 (13.1%)	2 (5.3%)
Lumpectomy	26 (68.4%)	6 (15.7%)	4 (10.5%)	2 (5.2%)
Mastectomy	12 (31.6%)	0	0	0
SLNB	33 (86.8%)	6 (15.7%)	4 (10.5%)	2 (5.2%)
ALND	5 (13.2%)	0	0	0
Age	56.5 (mean)/59 (median)	5 patients in post-menopause	5 (13.5%)	0
BMI	25.09 (mean)/24.42 (median)	2 patients with BMI > 25 experienced	1 (2.7%)	1 (2.7%)
Smokers	15 (40.5%)	4 (10.8%)	3 (8.1%)	1 (2.7%)
NACT	7 (18.9%)	0	0	0
Positive Lymph Nodes	9 (23.6%)	1 (2.6%)	1 (2.6%)	0

## Data Availability

The data presented in this study are available from the corresponding author upon request.

## Abbreviations

The following abbreviations are used in this manuscript:

SLNB	Sentinel lymph node biopsy
ALND	Axillary lymph node dissection
BCS	Breast-conserving surgery
NACT	Neoadjuvant chemotherapy
